# Molecular Profiling of Digestive Grade 3 Neuroendocrine Tumors Reveals a Shared Molecular Framework with Lower-Grade Tumors, Marked Heterogeneity, and Therapeutic Opportunities

**DOI:** 10.1007/s12022-026-09918-y

**Published:** 2026-05-12

**Authors:** Amedeo Sciarra, Laura Libera, Annarita Destro, Roberta Maragliano, Daniela Furlan, Vincenzo Guastafierro, Deborah Marchiori, Alessandro Vanoli, Raul S. Gonzalez, Igor Letovanec, Stefano Lazzi, Stefano La Rosa, Silvia Uccella

**Affiliations:** 1 Unit of Pathology, Department of Medicine and Technological Innovation, University of Insubria, Varese, Italy; 2 Histopathology, Central Institute, Valais Hospital, Avenue Grand Champsec 86, Sion, 1951 Switzerland; 3 Unit of Pathology, University Hospital (ASST Sette Laghi), Varese, Italy; 4 Department of Pathology, IRCCS Humanitas Research Hospital, Milan, Italy; 5 Department of Biomedical Sciences, Humanitas University, Milan, Italy; 6 Department of Molecular Medicine, University of Pavia, Pavia, Italy; 7 Unit of Anatomic Pathology, IRCCS San Matteo Hospital Foundation, Pavia, Italy; 8 Department of Pathology, Emory University Hospital, Atlanta, GA USA; 9 Institute of Pathology, Department of Medical Biotechnology, Siena University Hospital, Siena, Italy; 10 Hereditary Cancer Research Center, Department of Medicine and Technological Innovation, University of Insubria, Varese, Italy

**Keywords:** Gastroenteropancreatic neuroendocrine neoplasms, Neuroendocrine tumor, Neuroendocrine carcinoma, Transcriptomics, Genomics, Immunotherapy

## Abstract

Well-differentiated neuroendocrine tumors, grade 3 (NETs G3) of the digestive system are high-grade neuroendocrine neoplasms characterized by well-differentiated neuroendocrine morphology and high proliferative activity. Their molecular identity and therapeutic vulnerabilities remain incompletely defined. We performed a transcriptomic analysis and a comprehensive genomic profiling of 40 cases, including 26 NETs G3, 8 NETs G1/G2, and 6 neuroendocrine carcinomas (NECs). Targeted sequencing assessed alterations at both the DNA and RNA level, and gene expression profiling was conducted using a targeted immune-oncology panel. NETs G3 retained recurrent alterations in chromatin-regulatory genes characteristic of well-differentiated tumors and largely lacked the consistent *TP53* and *RB1* inactivation typical of NECs. Transcriptomic and genomic analysis demonstrated separation of NECs from NETs, with NETs G3 partially overlapping with NETs G1/G2 supporting a model of molecular relatedness. However, when compared with NETs G1/G2, NETs G3 showed upregulation of extracellular matrix remodeling, metabolic reprogramming, and cytokine signaling pathways. NETs G3 were heterogeneous in terms of number of genomic alterations per case and involved genes. A distinctive genomic feature was the predominance of large-segment copy-number losses, frequently involving chromatin-regulatory regions on chromosomes X and 10. Potentially targetable alterations were detected in up to 25% of NETs G3. Analysis of immune microenvironment supported a poorly inflamed tumor status, with individual cases harboring microsatellite instability or enrichment in tumor-infiltrating lymphocytes potentially amenable to immunotherapy.

**Supplementary Information:**

The online version contains supplementary material available at 10.1007/s12022-026-09918-y.

## Introduction

The current World Health Organization (WHO) classification of digestive neuroendocrine neoplasms (NENs) [1] is based on morphological differentiation and proliferative activity. According to this framework classification, the spectrum of neuroendocrine neoplasms (NENs) encompasses neuroendocrine tumors (NETs) - which are further graded from G1 to G3 according to mitotic count and Ki-67 proliferation index - and neuroendocrine carcinomas (NECs), which are high-grade neoplasms by definition. NETs G3 share morphological features with lower-grade NETs despite showing a proliferative activity overlapping with NECs [2]. From a clinical and prognostic point of view, NETs G3 appear more aggressive than NETs G1/G2 but have a significantly better prognosis than NECs [3].

Molecular studies, predominantly on pancreatic NENs, have shown that NETs harbor recurrent *MEN1*, *DAXX*, and *ATRX* mutations and frequent alterations in the chromatin remodeling and PI3K pathways. Conversely, NECs are characterized by *TP53* and *RB1* mutations [4, 5]. Available evidence on digestive NETs G3 suggests that these tumors retain the mutational profile of NETs G1/G2, including *MEN1*, *ATRX*, and *DAXX* mutation, with a fraction of cases further presenting *TP53* mutations [6]. Moreover, compared to NECs, NETs G3 appear to exhibit lower tumor mutational burden (TMB) and infrequent *RB1* mutations, and they only exceptionally show microsatellite instability (MSI) [7–9]. The presence of MSI is clinically relevant for patient management, influencing prognosis, potential response to immunotherapy, and the need for genetic counselling if Lynch syndrome is suspected [10, 11]. Importantly, the genetic landscape of NET G3 does not seem to include other adenocarcinoma-like alterations that are, in contrast, commonly observed in digestive NEC [3]. These data underpin the evolving classification of digestive NENs and have practical diagnostic implications, including the use of ATRX, DAXX, p53, and RB1 immunohistochemistry to accurately distinguish NETs G3 from NECs [1].

The clinical behavior of NETs G3 is intermediate between those of NETs G1/G2 and NECs [12, 13] and, from a therapeutic standpoint, all treatment options available for advanced NETs (somatostatin analogues, peptide receptor radionuclide therapy, mTOR and RTK inhibitors, and capecitabine/temozolomide regimen) and NECs (alkylating based chemotherapy, immunotherapy) can be proposed to NET G3 patients [14]. The choice is essentially based on Ki-67 proliferative index, tumor stage, and predicted sensitivity to somatostatin analogues [15, 16]. Importantly, the role of immune checkpoint inhibitors (ICI) in NET G3 is limited, as most digestive NETs, including NETs G3, have been reported to exhibit an “immune-cold” phenotype [17]. Only a minority of cases, for example, show PD-L1 expression and a prominent intratumor T-lymphocytes (TILs) infiltration [18, 19]. However, additional data are required to identify biomarkers for target treatment and to develop a specific, individualized approach for NET G3 patients.

Given the need for additional data to characterize the molecular landscape of NETs G3, this study comprehensively analyzed the molecular features of a series of digestive NETs G3, compared with series of NETs G1/G2 and NECs. The aims were to delineate the extent of molecular overlap across these categories, define the internal heterogeneity and core biological features of NETs G3, and identify potentially targetable alterations as well as immunotherapy-relevant subsets.

## Materials and Methods

### Case Series

Formalin-fixed paraffin-embedded (FFPE) tissues from digestive NENs were collected through an international collaborative group including centers in Italy, Switzerland, and the United States. Three independent expert pathologists centrally reviewed hematoxylin–eosin and available immunohistochemical stains (AS, SU, SLR). Cytology specimens, decalcified materials, archival blocks older than 15 years, and previously treated cases were excluded. The study included only pure NETs and NECs; mixed neuroendocrine-non neuroendocrine neoplasms (MiNENs) were excluded.

Diagnoses were confirmed or updated according to the most recent WHO Classification of Digestive System Tumors, with discrepancies resolved by consensus. Baseline analysis included morphology reappraisal, immunohistochemical evaluation of at least two neuroendocrine differentiation markers (e.g., synaptophysin, chromogranin, INSM1) and somatostatin receptor 2 A (SSTR2A), scored according to validated criteria [20]. Mitotic count and Ki-67 (clone MIB1) proliferation index were re-assessed on digital images or printouts of camera-captured images of areas of highest proliferative activity (in regions containing 500–2000 tumor cells). Samples were required to have adequate histologic quality, tumor cellularity ≥ 20%, and limited necrosis (< 50%), with microdissection performed when needed.

### Gene Expression Profiling

Gene expression analysis was performed using the nCounter PanCancer IO 360 Panel (NanoString Technologies, WA, USA), which quantifies 750 cancer- and immunity-related genes and 20 housekeeping genes, including validated immune-related signatures such as the 18-gene Tumor Inflammation Signature (TIS). RNA was extracted using the Maxwell^®^ RSC RNA FFPE Kit (Promega) and quantified using the Qubit™ RNA XR Assay (Thermo Fisher Scientific, Waltham, MA, USA). 100–300 ng RNA per sample was used. Hybridization with reporter and capture probes was performed for 16–20 h at 65 °C, and samples were processed on the NanoString nCounter preparation station and scanned using the nCounter Digital Analyzer. A reference standard was run in parallel to correct technical variability. Raw counts were processed using nSolver™ software (v4.0.70) with the Advanced Analysis Module (v2.0.134). Normalization was performed using positive controls and the geometric mean of housekeeping genes, followed by background subtraction based on negative controls. Differential expression, pathway enrichment, and immune cell profiling were conducted using the NanoString Advanced Analysis tools. Unsupervised hierarchical clustering and principal component analysis (PCA) were applied to assess transcriptional heterogeneity and identify clusters across diagnostic categories (NET G1/G2, NET G3, and NEC). Pathway modulation was categorized according to the number of genes involved; magnitude and concordance of gene expression changes, and pathway activity shifts were calculated as mean log2 fold-change across significant genes within each pathway.

### Comprehensive Genomic Profiling

Targeted DNA and RNA sequencing was performed using the Oncomine Comprehensive Assay (OCA) Plus (Thermo Fisher Scientific), enabling detection of single nucleotide variants (SNVs), indels, copy number alterations (CNAs), chromosomal alterations, loss of heterozygosity (LOH), MSI markers, genomic instability metrics (GIM), and gene fusions across a panel covering hotspot regions of 517 cancer-related genes (1.4 Mb). Genomic DNA and RNA were extracted using Maxwell RSC DNA and RNA FFPE kits (Promega) and quantified using Qubit assays. Nucleic acid integrity was assessed using TapeStation 4200 (Agilent). Samples not meeting minimum quality thresholds were excluded. Libraries were prepared from 20 ng DNA and 20 ng RNA following the manufacturer’s workflow. DNA libraries were generated by enzymatic fragmentation, adapter ligation, and PCR enrichment; RNA libraries were prepared through reverse transcription and targeted amplification of fusion transcripts. Libraries were pooled, templated on the Ion Chef System, and sequenced on the Ion S5 XL platform using Ion 540 chips. Data processing, alignment (hg19), and variant calling were performed using Torrent Suite (v5.12) and Ion Reporter (v5.16). Variants were annotated and filtered using standard quality parameters, and CNAs were inferred using internal normalization and validated read-depth thresholds. The total number of detected SNVs/indels/CNAs was used for comparative purposes. Actionable variants were interpreted using OncoKB and ClinVar annotations.

### Statistical Analysis

Variables were reported as numbers and percentages and summarized as median with range or frequency and percentage. Comparisons were performed using the Mann–Whitney U, Kruskal–Wallis χ2, and Fisher exact tests as appropriate. The degree of overlap between sets of differentially expressed genes (DEGs) obtained from each pairwise comparison was quantified using the Jaccard similarity index (J), defined as the ratio between the number of shared genes and the total number of unique genes across sets. Values ranged from 0 (no overlap) to 1 (complete overlap) and were assessed for enrichment with a hypergeometric test. Tests were two-sided, with a significance level of 0.05. Raw (unadjusted) *p* < 0.05 was used in differential gene expression analysis. Analysis was performed with SPSS 26.0 (^®^2013 SPSS Inc., Chicago, IL, USA).

## Results

This study assembled a cohort of 40 digestive NENs, including 26 NETs G3, 8 NETs G1/G2, and 6 NECs (5 of large and 1 of small cell type). Clinicopathological data are detailed in Table 1. NET G3 patients had a median age of 56 years (range 15–78), similar to NET G1/G2 (58 years, range 50–81), whereas NECs occurred in significantly older individuals (78 years, range 38–83; *p* = 0.042). Sex distribution did not significantly differ, although NET G3 showed a male predominance (69%). Median tumor size was identical in NET G3 and NET G1/G2 (2.5 cm), while NEC tended to be larger (7.1 cm). Within the NET G3 group, the pancreas was the predominant primary site (15/26, 58%), followed by the ileum (4/26), colon (3/26), stomach (2/26), and single cases arising in the duodenum and rectum. Median Ki-67 index was 25% (21–65) in NETs G3, intermediate between NETs G1/G2 (2%, 1–10) and NECs (80%, 75–90) (*p* < 0.001) with no overlap between NETs G3 and NECs. Median mitotic count per 2 mm² was likewise intermediate in NET G3 (5, range 0–33) compared with NET G1/G2 (1, 0–11) and NEC (35, 3–64) (*p* = 0.001). Strong SSTR2A expression (score 3+) was observed in 91% of NETs G3, 100% of NETs G1/G2 and in one NEC (*p* < 0.001).

Transcriptomic profiling (NanoString IO360) generated valid results in 34 quality-controlled samples, corresponding to 22 NETs G3, 7 NETs G1/G2, and 5 NECs. Targeted DNA/RNA sequencing yielded exploitable data in 20 samples after quality control, including 13 NETs G3, 4 NETs G1/G2, and 3 NECs.

**Table 1:** Clinico-pathological baseline

Variable	Category	NET G3 *N* = 26	NET G1/G2 *N* = 8	NEC *N* = 6	*P*-value
Age	Median (range)	56 (15–78)	58 (50–81)	78 (38–83)	0.042
Sex	Male	18 (69%)	3 (38%)	3 (50%)	0.260
Size (cm, largest nodule)	Median (range)	2.5 (0.8–8.2)	2.5 (0.9-7)	7.1 (2–17)	0.383
Neoplasm origin	Pancreas	15 (58%)	1 (12%)	1 (17%)	0.002
	Small bowel	5 (19%)	6 (75%)	0	
	Colorectal	4 (15%)	1 (12%)	3 (50%)	
	Stomach	2 (8%)	0	2 (33%)	
Neoplasm site	Primary	23 (88)%	8 (100%)	6 (100%)	0.606
	Metastasis	3 (12)%*	0	0	
Sample type	Resection	26 (100)%	8 (100%)	6 (100%)	1
Ki-67 proliferation index	Median (range)	25% (21–65)	2% (1–10)	80% (75–90)	< 0.001
Mitotic count per 2mm^2^	Median (range)	5 (0–33)	1 (0–11)	35 (3–64)	0.001
SSTR2A expression	score 3	21** (91%)	8 (100%)	1 (17%)	< 0.001

*In one NET G3 metastasis the primary tumor was a NET G1

**Data missing for 3 NET G3 cases

### Molecular Positioning of NET G3 as Compared to NET G1/G2 and NEC

#### Gene Expression Profiling

**Fig. 1:**
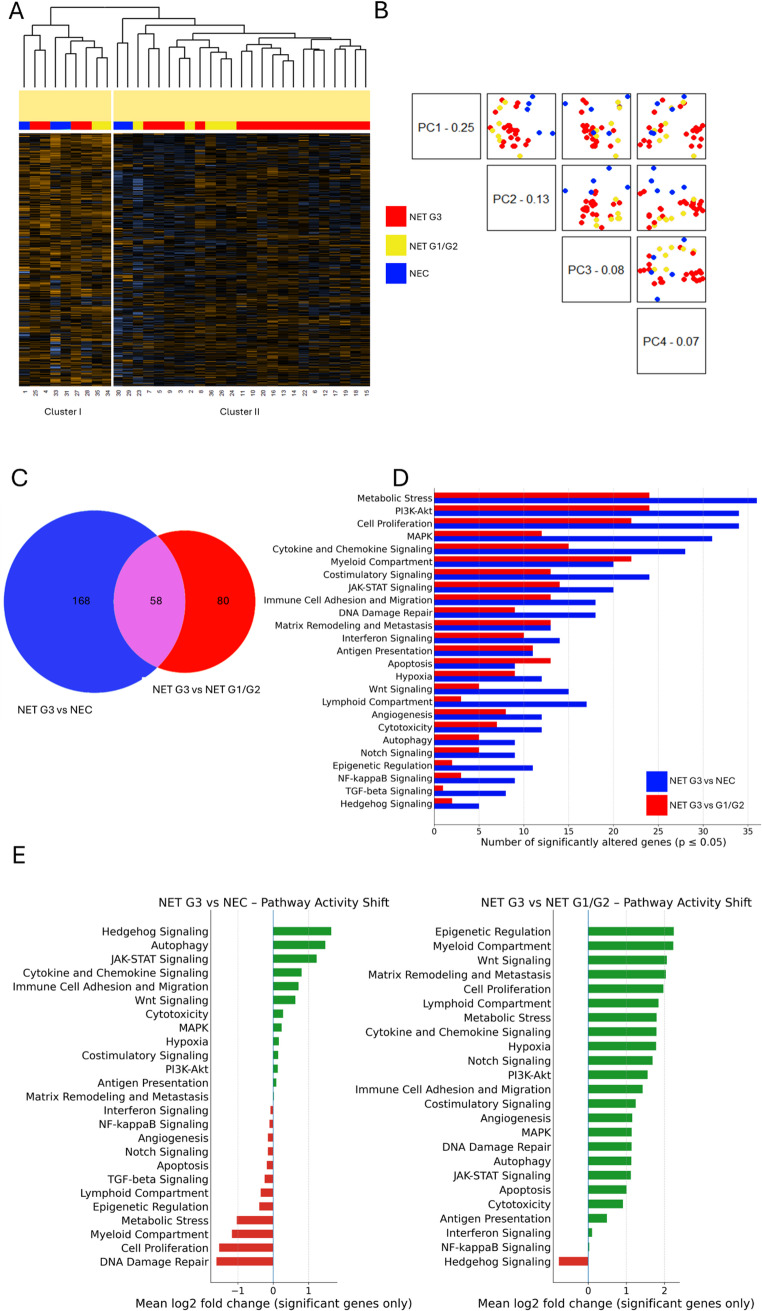
Transcriptomic profile of NETs G3 as compared to NETs G1/G2 and NECs. Legend: **A** Unsupervised hierarchical clustering identified two main clusters without clear segregation of NETs G3 from NETs G1/G2 or NECs. **B** Principal component analysis demonstrated that the first two principal components (PC1 and PC2) accounted for 38% of the total variance and segregated NETs (G1/G2 and G3) from NECs. **C** Venn diagram showed that only a minority (n = 58) of the differentially expressed genes were shared between the NET G3 vs NEC and NET G3 vs NET G1/G2 comparisons. **D** The number of differentially expressed genes was higher in the NET G3 vs NEC comparison across most evaluated pathways. **E** Pathway activity analysis revealed a heterogeneous pattern in NET G3 vs NEC, with both up- and downregulated pathways, whereas NET G3 vs NET G1/G2 comparison was characterized predominantly by pathway upregulation

Unsupervised hierarchical clustering analysis of IO360 genes separated neoplasms into two expression clusters (Fig. 1A). Cluster membership was not statistically associated with diagnostic categories, although most NETs G1/G2 and G3 grouped together (cluster II). Principal component analysis (PCA) confirmed separation of NECs from NETs, with no clear distinction between NETs G3 and NETs G1/G2 (Fig. 1B). The greatest transcriptomic divergence was observed between NETs G3 and NECs, where 226 genes were differentially expressed, as compared to the 138 differentially expressed genes between NETs G3 and NETs G1/G2 (Fig. 1C). The number of differentially expressed genes was also higher between NETs G3 and NECs than between NETs G3 and NETs G1/G2 in virtually all pathways (Fig. 1D). In addition, the pathway activity shift in NETs G3 compared to NECs presented a skewed profile, with several up- and downregulated pathways. In contrast, NETs G3 compared to NETs G1/G2 showed predominantly upregulated pathways, supporting a transcriptomic continuum between NET G1/G2 and G3 (Fig. 1E). In NET G3 vs NEC, the most pronounced negative shifts involved cell-cycle regulation and DNA-damage repair, together with metabolic-stress response, whereas positive shifts were observed in Hedgehog, JAK–STAT signaling, and autophagy. In NET G3 vs NET G1/G2, pathway modulation was predominantly positive, mainly involving extracellular-matrix remodeling, hypoxia/metabolic stress, and cytokine/JAK–STAT signaling, with only relevant negative shift in Hedgehog signaling. These trends are further illustrated in Fig. S1.

#### Targeted Sequencing

**Fig. 2:**
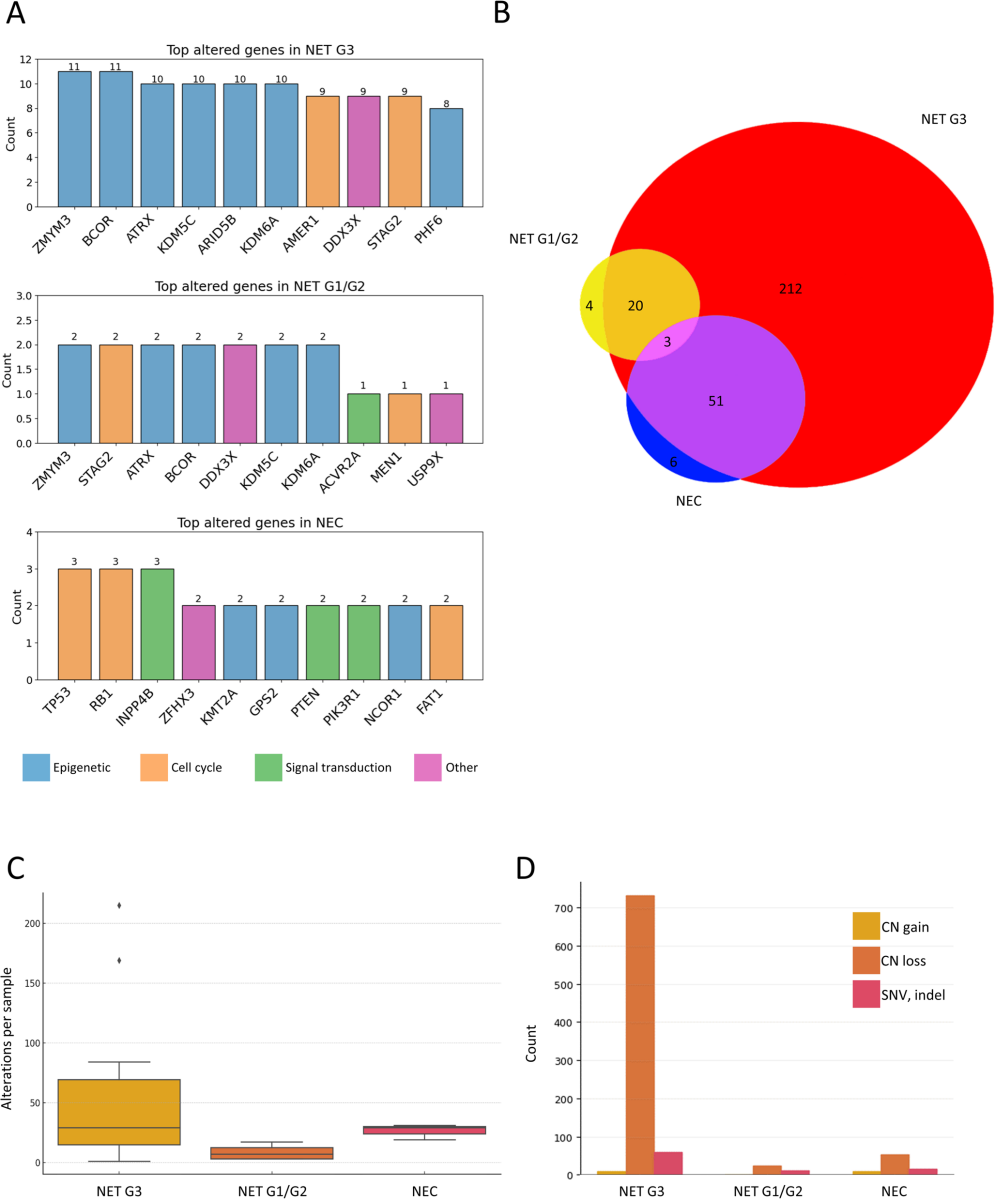
NET G3 as compared to NETs G1/G2 and NECs. Legend. **A** Targeted deep sequencing revealed that NET G3 and NET G1/G2 most frequently harbored alterations in genes involved in epigenetic regulation, whereas cell cycle–related alterations predominated in NEC. **B** Only a limited number of molecular alterations were shared across the three diagnostic categories, with NET G3 exhibiting the highest number of uniquely altered genes. **C** NET G3 showed a high median number and the widest range of genomic alterations per sample. **D** Copy number losses represented the most frequent type of alteration in NET G3

At the genomic level (next-generation sequencing analysis), the most frequent altered genes in both NETs G3 and NETs G1/G2 participated in epigenetic and chromatin control, with *ZYMM3*, *BCOR*, *ATRX*, *KDM6A*, and *KDM5C* among the most frequent 10 altered genes (Fig. 2A). In contrast, in NECs, tumor suppressor and cell cycle genes predominated. More broadly, NET G3 was overwhelmingly enriched in uniquely altered genes (*n* = 212), with limited overlap with both NET G1/G2 (23 genes) and NEC (54 genes) (Fig. 2B). Accordingly, a low J-index was observed in both comparisons (0.18 vs. NEC and 0.08 vs. G1/G2), underscoring low similarity. Indeed, NET G3 accounted for the majority of molecular alterations (798 events, including SNV, indels, and CNA affecting 286 genes), compared to NEC (79 events; 60 genes) and NET G1/G2 (34 events; 27 genes). Alteration burden per sample differed by category with borderline overall significance (Kruskal–Wallis *p* = 0.058), with a significant pairwise difference between NET G3 and NET G1/G2 (*p* = 0.033).

### NET G3 Heterogeneity and Core Characteristics

Across platforms, NETs G3 showed substantial heterogeneity. Transcriptomically, NETs G3 distributed across both unsupervised clusters (4/22 in Cluster I and 18/22 in Cluster II, Fig. 1A), indicating internal variability despite a common diagnostic label. The overlap of differentially expressed genes among NET G3 vs. NEC and G1/G2 was limited, as only 58 genes overlapped (hypergeometric *p* = 0.0398) (Fig. 1C). Moreover, only 15/58 genes were deregulated in the same direction.

NETs G3 showed a high number of genomic alterations (median 29, range 1-215) as compared to NETs G1/2 (median 7, range 3–17) and NECs (median 29, range 19–31) and were highly heterogeneous in terms of number of alterations per sample (Fig. 2C), dominated by copy number loss (Fig. 2D).

**Fig. 3:**
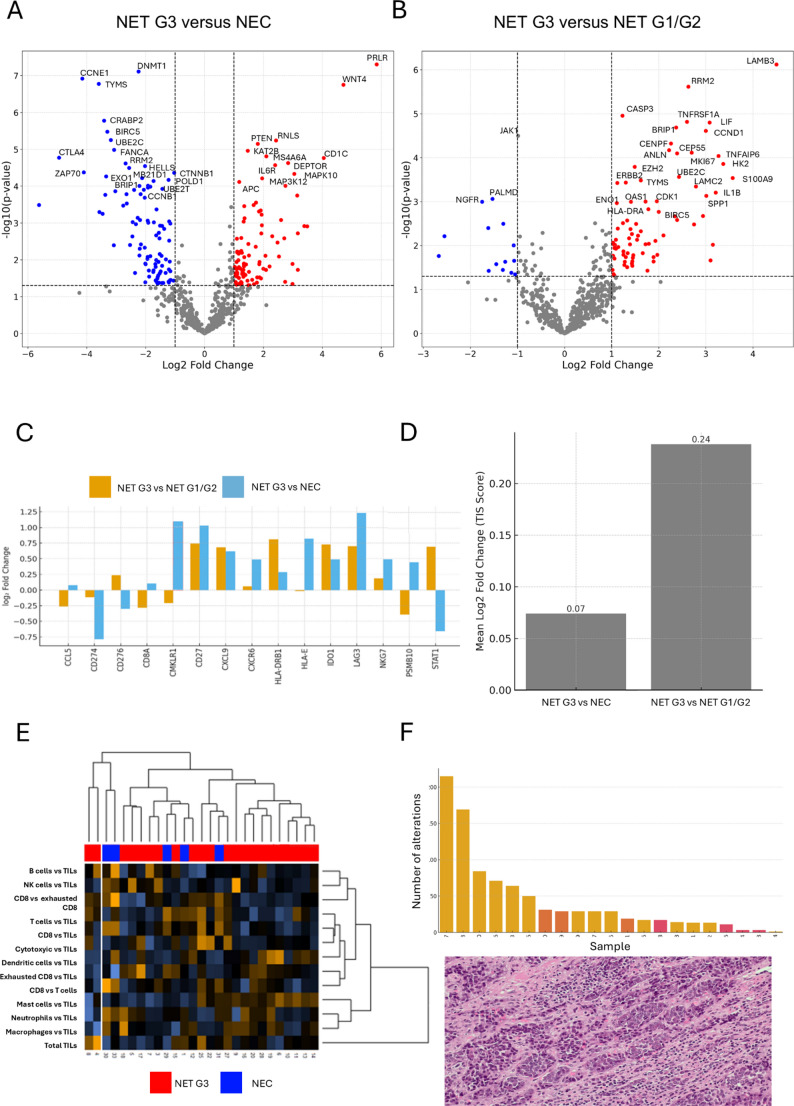
Differential gene expression analysis of NETs G3 as compared to NETs G1/G2 and NECs. Legend: **A**, **B** Volcano plots depicting significantly upregulated (red) and downregulated (blue) genes in the NET G3 vs NEC (**A**) and NET G3 vs NET G1/G2 (**B**) comparisons. **C** Focused analysis of genes included in the Tumor Inflammation Signature (TIS) demonstrated low-magnitude and predominantly non-significant expression differences among NET G3, NET G1/G2, and NEC. **D** The composite TIS score was low in both comparisons. **E** Immune cell signature analysis demonstrated a predominantly low-to-intermediate tumor-infiltrating lymphocyte (TIL) profile in NET G3, with a small subset of cases exhibiting a TIL-enriched pattern. **F** TIL-enriched case 4 corresponded to a cecal pediatric NET G3 characterized by a low genomic alteration burden and prominent lymphocytic infiltration on conventional H&E histology

Despite the global molecular heterogeneity of NET G3, several reproducible group-level features were identified. Transcriptomically, in comparison with NECs, NETs G3 showed significant upregulation of genes involved in hormone signaling and morphologic control, including *PRLR* (log₂FC = + 5.85, *p* < 0.0001) and *WNT4* (log₂FC = + 4.72, *p* < 0.0001), suggesting preserved well-differentiated signature (Fig. 3A). Against NETs G1/G2, NETs G3 showed positive pathway shifts in extracellular-matrix remodeling, metabolic reprogramming, and cytokine/JAK–STAT signaling (Fig. 1E). Indeed, among the most significantly upregulated transcripts were *LAMB3* (log₂FC = + 4.50, *p* < 0.0001), a basal membrane laminin, and *TNFAIP6* (log₂FC = + 3.27, *p* = 0.0001), a key regulator of extracellular matrix interactions; *CASP3* (log₂FC = + 1.23, *p* < 0.0001), *LIF* (log₂FC = + 3.08, *p* < 0.0001), and *IL1B* (log₂FC = + 3.21, *p* = 0.0006) that are target and regulatory genes of the JAK–STAT–MAPK signaling; and the glycolytic enzyme *HK2* (log₂FC = + 3.37, *p* = 0.0001). Similarly, *EZH2*, an epigenetic regulator associated with proliferative activity, was significantly upregulated in NET G3 compared with NET G1/G2 (log₂FC = + 1.49, *p* = 0.0002), while showing opposite regulation in the NET G3 versus NEC comparison (log₂FC = − 1.84, *p* = 0.0001) (Fig. 3B).

At the genomic level, NETs G3 showed alterations (SNVs, indels, CNAs) in epigenetic and chromatin control genes *MEN1*, *DAXX*, and *ATRX* in 5/13 cases, as compared to 2/4 of NETs G1/G2 and no NECs, underscoring a common neuroendocrine backbone profile of NETs independent from grade. Notably, *ATRX* alterations were not restricted to pancreatic primaries but also included a duodenal NET G3. Indeed, genes involved in cell replication network were significantly downregulated in NETs G3, as compared to NEC. These genes included *RRM2* (log₂FC = − 2.68, *p* < 0.0001), *TYMS* (log₂FC = − 3.59, *p* < 0.0001), both involved in synthesis of DNA precursors, and *DNMT1* (log₂FC = − 2.24, *p* < 0.0001), implicated in DNA methylation of subtelomeric regions (Fig. 3A).

In contrast, *TP53* and *RB1* alterations were a key feature of NECs (3/3 and 2/3 cases, respectively) as compared to NETs G3 (*p* = 0.002), where *TP53* mutation alone was detected in a minority (2/13) of NETs G3 and *RB1* was always intact. No *TP53* and *RB1* alterations were identified in G1/G2 NETs.

A key feature of NETs G3 in this dataset was the presence of frequent CN losses (average CN loss rate 64; range 6–207), occurring in specific chromosomal regions, recognized as recurrent large-segment deletions. Indeed, 8/13 (62%) NETs G3 presented recurrent deletions involving Xp22.33–p11.22 and Xq11.2–q28 loci, which contain several chromatin-regulators (*KDM5C*, *KDM6A*, *BCOR*, *ZMYM3*, *DDX3X*, *STAG2*, *CUL4B)*, while 6/13 (46%) NETs G3 presented recurrent deletions involving the 10q11.21–q26.13 locus, which contains the histone demethylase *ARID5B*. This Xp/Xq deletion pattern was highly specific for NET G3 (specificity 97.6%; *p* = 0.0001 vs NET G1/G2 and NEC).

In addition, NETs G3 were also characterized by the presence of frequent PI3K/mTOR pathways mutations (*PIK3CA*, *PTEN*, *TSC2*, detected in 21% of cases). This observation was combined with the selective up-regulation of downstream metabolic and cytokine-signaling genes as compared to NETs G1/G2 at gene expression profiling. Also, one duodenal case demonstrated *VHL* mutation, and a global increased expression of *LAMB3* and *S100A9*, involved in hypoxia-linked matrix remodeling, was observed in the NET G3 vs NET G1/G2 comparison.

### Targetable Alteration and Immunotherapy Susceptibility in NET G3

According to the OncoKB database, targetable mutations were detected in 3 out of 13 (23%) NETs G3, namely *ARID1A* p.Gln524AlafsTer94, *PIK3CA* p.His1047Arg and *PTEN* p.Gly165Ar (Table 2).

**Table 2:** Pathogenic, likely pathogenic, and targetable mutations in NETs G3 according to the OncoKB database

Sample	Gene	Protein change	AF%	OncoKB level	Drug	Indication
5	*TP53*	p.Arg282Trp	80			
5	*ARID1A*	p.Gln524AlafsTer94	43	4	PLX2853, Tazemetostat	all solid tumors
6	*PIK3CA*	p.His1047Arg	12	1	Capivasertib + Fulvestrant	Breast cancer
				4	AZD8186, GSK2636771	Breast cancer
6	*MEN1*	p.?	50			
6	*TSC2*	p.?	57			
12	*TP53*	p.Cys135Ter	57			
13	*CDKN1B*	p.Phe87SerfsTer32	23			
18	*VHL*	p.Arg161Gln	70			
18	*PTEN*	p.Gly165Arg	63	1	Capivasertib + Fulvestrant	Breast cancer
				4	AZD8186, GSK2636771	Breast cancer
18	*ATRX*	p.Phe2113SerfsTer9	83			
18	*MEN1*	p.Arg451ProfsTer3	72			
21	*KMT2B*	p.Val2334SerfsTer105	98			
28	*MGA*	p.Cys1270ValfsTer21	8			
37	*MEN1*	p.Trp188Cys	80			
37	*MSH2*	p.Arg680Ter	83			

Concerning the homologous recombination deficiency assessment, no case reached a formal genomic-instability threshold as defined by the GIM score, and recombination repair gene alteration counts did not differ across groups. Nevertheless, 61 losses and 2 SNVs of unknown significance were detected in HRR genes of NET G3 cases.

Regarding microsatellite instability, one NEC case was MSI, as was already known from the diagnostic workup, while one NET G3 carried an *MSH2* p.Arg680Ter83 mutation, which was annotated as pathogenic for germline classification (Lynch Syndrome) in the ClinVar database.

For immune transcriptomics, the TIS score supported the notion of low immune activation. When individual TIS genes were analyzed in NET G3 vs NEC and NET G1/G2, none reached significance except for *CMKLR1* (*p* = 0.011 for NET G3 vs. NEC), and the fold changes were low (Fig. 3C). The aggregate TIS scores were also low, namely 0.07 (NET G3 vs NEC) and 0.24 (NET G3 vs NET G1/G2) (Fig. 3D). When looking at immune cell signatures, NET G3 generally showed a low to intermediate TILs signature, with a notable subset of 2 cases with a TILs enriched profile (Fig. 3E). Complementary PCA and PLS-DA analyses of immune cell profiling scores suggested differences between NET G3 and NEC (Fig. S2). It is worth noting that one of these cases (case 4) occurred in a pediatric patient that had a cecal NET G3 with the lowest burden of genomic alterations and several TILs on conventional morphology (Fig. 3F).

Finally, no gene fusion was observed in the cohort studied.

## Discussion

This integrated genomic and transcriptomic analysis refines the molecular identity of digestive NET G3 within the spectrum of neuroendocrine neoplasms, in the frame for the current WHO classification [2], and provides biologically grounded insights relevant to precision medicine strategies.

At the genomic level, NECs were characterized by the expected *TP53* and *RB1* alterations, consistent with checkpoint failure and high proliferative drive described in previous series [4, 21]. In contrast, NETs G3 lacked *RB1* alterations, while showing recurrent mutations in chromatin-remodeling genes, already reported in NETs [22], including *MEN1*, *DAXX*, and *ATRX*. The low Jaccard similarity index between NET G3 and NEC, together with the lack of shared recurrent alterations, supports the concept that NET G3 represents a state of molecular continuity within the NET family rather than a morphologically deceptive NEC variant [8, 12]. These findings align with previous studies demonstrating that *TP53*/*RB1* inactivation defines NEC, whereas *MEN1*/*DAXX*/*ATRX*-driven chromatin dysregulation characterizes NETs across grades [4, 21–23], further quantifying the limited degree of molecular convergence between NET G3 and NEC.

Nevertheless, our results suggest that NETs G3 are not merely proliferative amplifications of G1/G2 NETs. While NET G3 retained the chromatin-regulatory backbone characteristic of well-differentiated neuroendocrine tumors, transcriptomic analyses also revealed selective upregulation of genes involved in extracellular matrix remodeling, cytokine/JAK–STAT signaling, and metabolic processes, including glycolysis (e.g., *HK2*). These findings support a model in which grade-associated differences reflect coordinated transcriptional changes rather than direct activation of specific oncogenic pathways. In this context, pathway-level enrichment should be interpreted cautiously, as it does not imply causality. In particular, enrichment in cytokine/JAK–STAT, angiogenesis, and metabolic pathways reprogramming [8, 24] may represent either primary biological processes or secondary downstream effects related to tumor progression and microenvironmental interactions. Therefore, these pathway alterations should be considered descriptive of the NET G3 transcriptional state, and further functional studies will be required to determine their mechanistic and therapeutic relevance.

*TP53* mutations were infrequent in NETs G3 (15%) and, in isolation, did not appear to be associated with loss of well-differentiated morphology or extreme proliferative activity, suggesting a state of molecular progression towards a high-grade neoplasm, rather than full NEC transformation. Although acquisition of *TP53* mutations (with or without *RB1*) has been associated with abrupt clinical progression in prior series [25, 26], the present data suggest that *TP53* alterations alone are insufficient to confer a NEC-like phenotype. These findings reinforce the biological distinction between NET G3 and NEC and support a cautious morphology-driven diagnostic approach, restrained use of isolated molecular markers, and consideration of tailored surveillance and therapeutic strategies. Notably, no cases in our cohort demonstrated concurrent *TP53* and *RB1* alterations or a fully NEC-like molecular phenotype, arguing against the presence of clearly identifiable “NEC-like NET G3” cases in this series. Differences across grades were also associated with progressive downregulation of Hedgehog signaling, which owns an established role in developmental patterning, tumor microenvironment and progression [27], and epigenetic dysregulation. For instance, *EZH2*, an epigenetic regulator reported to be associated with increased proliferative activity and p53 altered expression in digestive NETs [28], was upregulated in G3 vs G1/G2 NETs and downregulated in G3 vs NEC comparisons. However, it should be pointed that these observations are based on cross-sectional comparisons and therefore do not establish a stepwise progression between NET G1/G2 and NET G3.

A central feature of NETs G3 was the presence of a high burden of chromatin and epigenetic alterations, a feature of therapeutic interest, as studies challenging regimens targeting these alterations are ongoing [29]. Copy-number losses frequently involved large chromosomal segments encompassing regulators such as *KDM5C*, *KDM6A*, *BCOR*, and *ATRX*, genes essential for histone methylation balance and chromatin stability [22, 30]. This pattern suggests a model of chromatin-driven genomic instability distinct from the *TP53*/*RB1*-driven collapse typical of NEC [31]. A similar epigenetic-driven progression model was proposed in small intestinal NET G3, where grade escalation occurred without acquisition of recurrent new driver mutations [9]. In contrast to the limited spatial and temporal heterogeneity reported in that series, our cohort showed greater inter-case genomic variability, possibly reflecting site-related biological differences, particularly the predominance of pancreatic primaries. Notably, *ATRX* alteration was also identified in a duodenal NET G3, consistent with reported molecular overlap between duodenal and pancreatic NETs [32, 33].

In NET G3, genomic instability appears to arise primarily from disruption of epigenetic control rather than checkpoint abrogation. Concomitant transcriptional suppression of DNA repair genes and altered histone modification pathways suggests that epigenetic dysfunction may generate a permissive environment for replication stress and progressive structural alteration accumulation. Chromatin destabilization coexisted with transcriptional upregulation of genes involved in metabolic and cytokine signaling networks (e.g., *HK2*, *LIF*, *IL1B*). Similar metabolic reprogramming has been described in aggressive NET subsets and associated with grade progression [24]. These findings should be interpreted cautiously, as pathway enrichment analyses do not establish whether these signals represent primary oncogenic drivers or secondary downstream adaptations. More broadly, the pathway-level differences observed between NET G3, NET G1/G2, and NEC should be interpreted as reflecting coordinated transcriptional states rather than causally defined biological mechanisms. In particular, enrichment in cytokine/JAK–STAT signaling, extracellular matrix remodeling, and metabolic pathways may represent either primary processes contributing to tumor biology or secondary responses to tumor progression and microenvironmental interactions. Functional studies will be required to disentangle these possibilities. Recent spatial transcriptomic work in NEC and mixed adenocarcinoma-NEC/MiNEN has likewise highlighted transcriptional heterogeneity within NEC components, including subclusters not apparent on morphology alone, further supporting the concept that high-grade neuroendocrine neoplasms may harbor biologically relevant intratumoral diversity [34, 35]. The recurrent deletions of Xp and Xq regions introduce an additional biological dimension. Several genes located within these loci escape X-inactivation and are expressed biallelically in female cells. X-linked chromatin regulators such as *KDM6A* and *KDM5C* are known to escape inactivation and play tumor-suppressive roles in multiple cancer types. Loss of such regions may disproportionately affect male patients, who lack a compensatory second allele, suggesting a potential mechanistic explanation for the male predominance observed in this NET G3 series [36].

Although homologous recombination repair genes were frequently affected by copy-number losses in NET G3, no case met formal criteria for homologous recombination deficiency. Nonetheless, the enrichment of HRR-related alterations, together with *TP53*, *ATRX*, and *DAXX* involvement, remains mechanistically relevant [37], as these genes cooperate in alternative lengthening of telomeres (ALT) and genomic instability in pancreatic NETs [23, 30]. MSI was rare, consistent with previously published data in digestive NET G3 [8, 10]. The identification of a single NET G3 case harboring a pathogenic *MSH2* mutation reinforces the exceptional nature of MSI in NET G3 and suggests immunotherapy indications on individual cases. Immune transcriptomic analyses confirmed a predominantly “immune-cold” phenotype, in agreement with studies reporting absent or low PD-L1 expression and limited T-cell–inflamed signatures in most NETs, including G3 cases [18, 19, 38]. The observation that a NET G3 with high TILs lacked extensive chromatin-regulator losses raises the possibility that epigenetic disruption may contribute to immune exclusion. This hypothesis is consistent with growing evidence that chromatin state and methylation loss influence immune visibility [39], a report indicating that higher-grade PanNETs progressively lose interferon and chemokine expression [40], and data from a small intestinal high-grade NETs series, showing globally low tumor inflammation signatures [9]. Moreover, this observation is consistent with the limited activity of immune checkpoint inhibitor observed in unselected NET trials [9, 41, 42].

From a translational standpoint, potentially actionable alterations were identified in approximately one quarter of NETs G3. Recent precision oncology efforts in neuroendocrine neoplasms emphasize that actionable targets are present in a subset of tumors but require comprehensive profiling for detection [24, 43, 44]. When integrated with SSTR2A expression and Ki-67 index, molecular profiling may refine therapeutic stratification. An integrated molecular classification framework, implemented through multidisciplinary molecular tumor boards, may enhance patient stratification and inform individualized treatment.

### Limitations

This study has several limitations. First, the number of NEC cases is limited, which may reduce statistical power and limit the ability to capture the full molecular heterogeneity of this group. In addition, histological subtypes of NEC (small cell versus large cell) were not analyzed separately, and their potential transcriptional differences could not be assessed. Second, the retrospective and multi-institutional design resulted in incomplete clinical annotation for some cases, including prior tumor history. Third, the absence of matched primary and metastatic samples or longitudinal data precludes inference of tumor evolution or lineage progression. Therefore, our findings should be interpreted as reflecting molecular relatedness and continuity across NET grades rather than direct evidence of evolutionary trajectories.

## Supplementary Information

Below is the link to the electronic supplementary material.

Supplementary Material

## Data Availability

Data are accessible on request.
