# Supplementary material for: Molecular Profiling of Digestive Grade 3 Neuroendocrine Tumors Reveals a Shared Molecular Framework with Lower-Grade Tumors, Marked Heterogeneity, and Therapeutic Opportunities
Source: Endocr Pathol. 2026 May 12;37(1):23. doi: 10.1007/s12022-026-09918-y (PMC13167899; doi:10.1007/s12022-026-09918-y)
Supplement: Supplementary file 1 — Supplementary Material [file 12022_2026_9918_MOESM1_ESM.docx]

**Supplementary material**

[**Figure S1. Pathway activity trends across neuroendocrine neoplasms.** 2](#_Toc226455458)

[**Figure S2. Unsupervised and supervised dimensionality reduction of immune cell profiling data.** 3](#_Toc226455459)

# **Figure S1. Pathway activity trends across neuroendocrine neoplasms.**


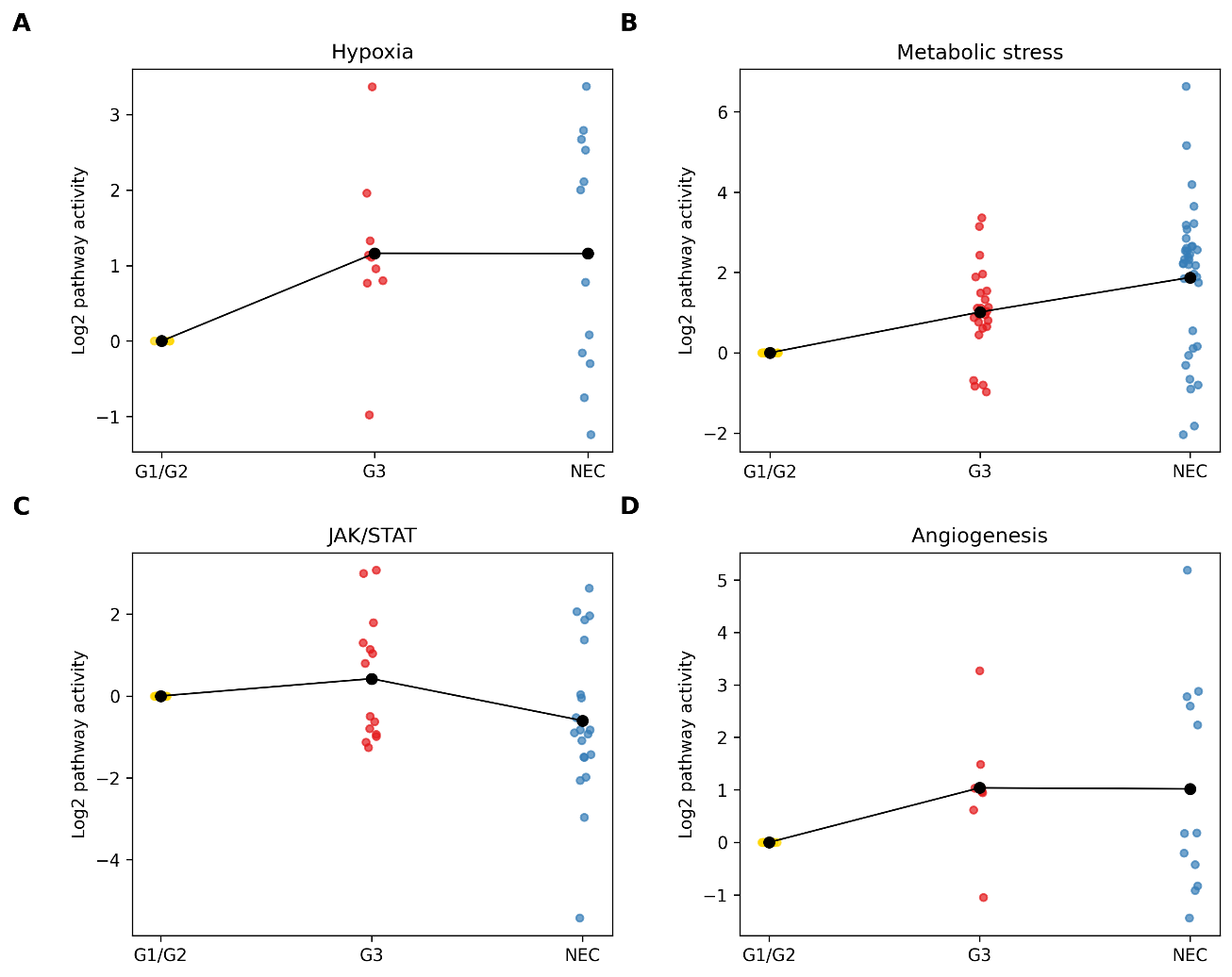


Legend: (A) Hypoxia-related signals peaked in NET G3. (B) Metabolic stress–associated signals showed a progressive increase across tumor categories. (C) JAK/STAT signaling showed an increase from NET G1/G2 to NET G3 modulation and was downregulated in NEC. (D) Angiogenesis-related signals increased from NET G1/G2 to NET G3 and plateaued in NEC. Pathway activity trends across neuroendocrine neoplasms. Pathway activity was calculated as the mean log₂ fold-change of significantly deregulated genes (p < 0.05) within each pathway (NanoString IO360 panel), reconstructed across NET G1/G2, NET G3, and NEC using pairwise comparisons. Dots represent individual gene-level log₂ fold-change values included in the pathway calculation.

# **Figure S2. Unsupervised and supervised dimensionality reduction of immune cell profiling data.**


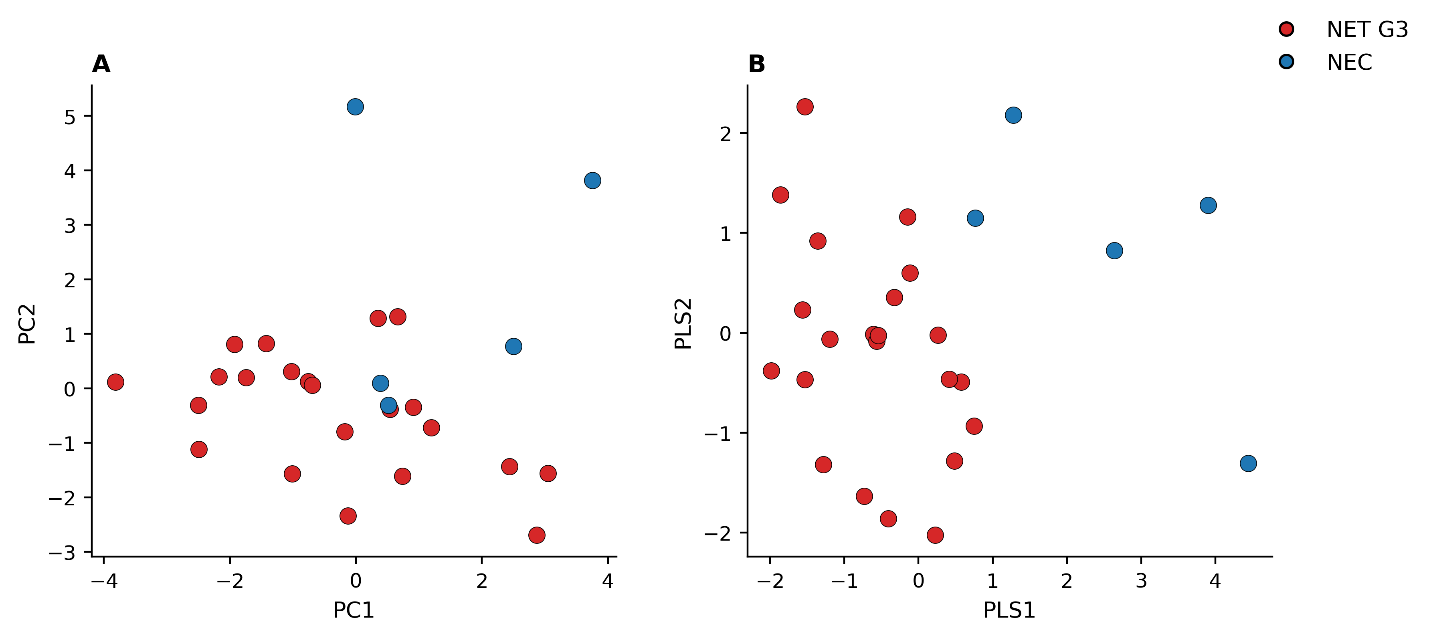


(A) Principal component analysis (PCA) based on relative cell type profiling scores derived from NanoString analysis provides an unsupervised overview of immune microenvironment variation across samples. Each point represents an individual tumor, colored according to tumor type (NET G3 vs NEC).

(B) Partial least squares discriminant analysis (PLS-DA) was performed using the same immune cell-type features with tumor type as the grouping variable. In contrast to PCA, PLS-DA is a supervised method that identifies latent components (PLS1 and PLS2) optimized to maximize separation between predefined groups.

Together, these complementary approaches show that while unsupervised analysis reveals overall variation in immune composition, supervised dimensionality reduction highlights clearer distinctions between NET G3 and NEC, supporting differences in immune microenvironment profiles. Results from PLS-DA should be interpreted as indicative of structured differences rather than definitive classification.
